# Supplementary material for: Explant Analysis of Utah Electrode Arrays Implanted in Human Cortex for Brain-Computer-Interfaces
Source: Front Bioeng Biotechnol. 2021 Dec 7;9:759711. doi: 10.3389/fbioe.2021.759711 (PMC8688945; doi:10.3389/fbioe.2021.759711)
Supplement: Supplementary file 1 [file DataSheet1.pdf]

Supplemental

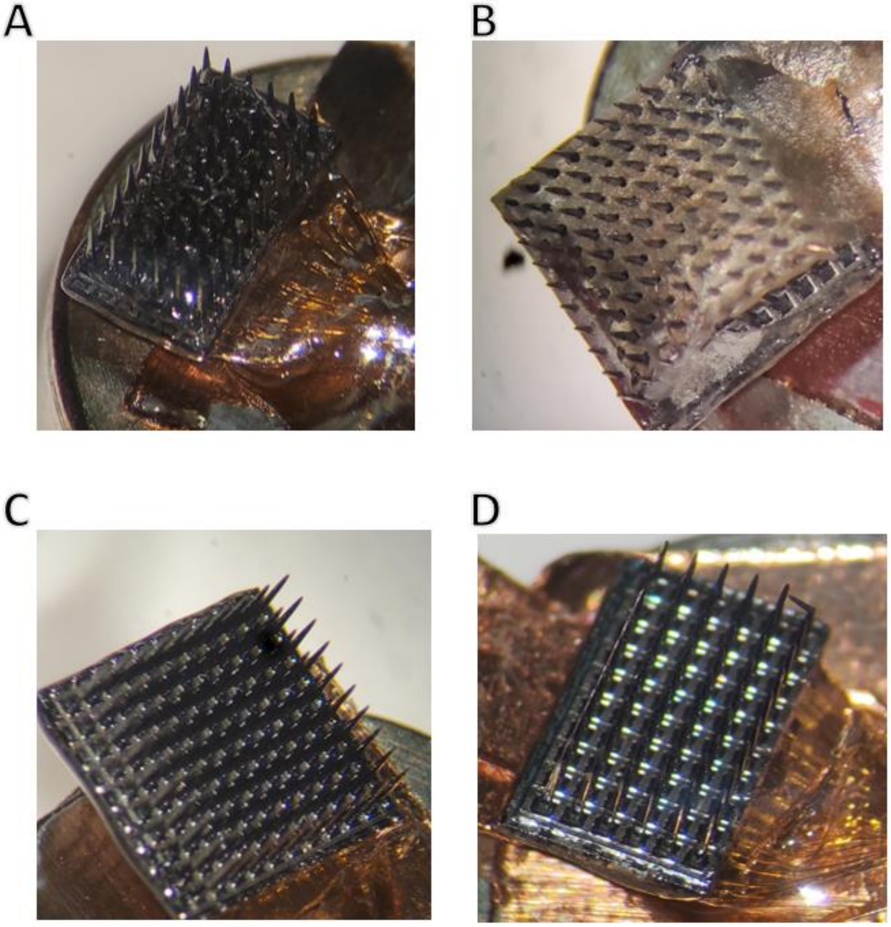

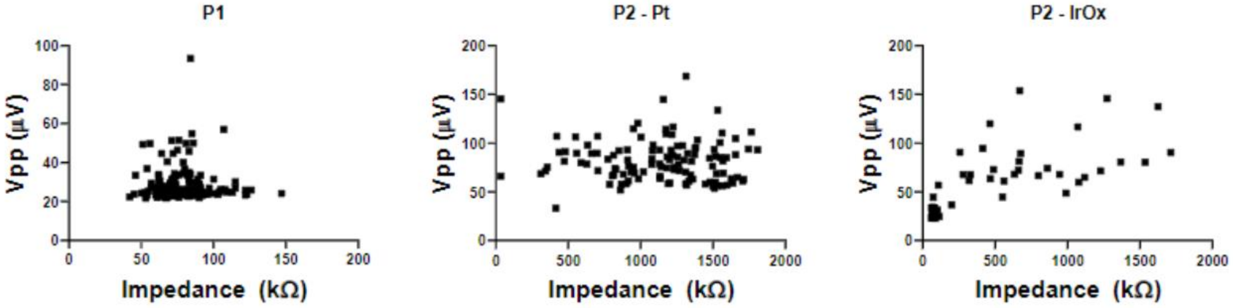
P1
P2 - Pt
P2 - IrOx
Vpp (µV)
Impedance (kΩ)
0
50
100
150
200
0
20
40
60
80
100
500
1000
1500
2000

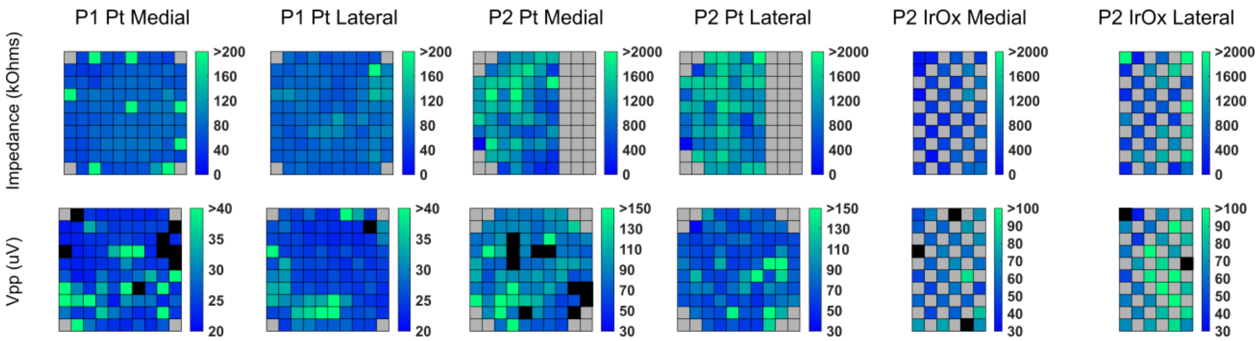

Supplemental Figure 1 Optical images P2 arrays: A) Medial stimulating array from P2. B) Medial recording array from P2. C) Lateral recording array from P2. D) Lateral stimulating array from P2.

Supplemental figure 2: Comparisons of impedance and Vpp for P1, P2 platinum electrodes, and P2 IrOx electrodes on the last day of recording. No trends between impedance and Vpp were observed for either of the platinum tipped recording arrays.

Supplemental Figure 3: Impedances and Vpp for individual electrodes on the last day of recording. Lower impedances were observed on the arrays implanted in P1 due to the length of implantation. Impedances were not measured on every electrode in P2 due to hardware limitations, namely that the impedance cable could only measure impedances from a total of 96 channels across two arrays. Colors are linearly scaled.
